# Supplementary material for: Splice-Junction-Based Mapping of Alternative Isoforms in the Human Proteome
Source: Cell Rep. Author manuscript; Available in PMC 2020 Jan 15. (PMC6961840; doi:10.1016/j.celrep.2019.11.026)
Supplement: 2 [file NIHMS1546469-supplement-2.zip › DF1/PXD010154/Testis/Testis_27_MAP4_MYHDDDLADLVFPSSATADTSIFAGQNDPLKDSYVPLELAK.pdf]

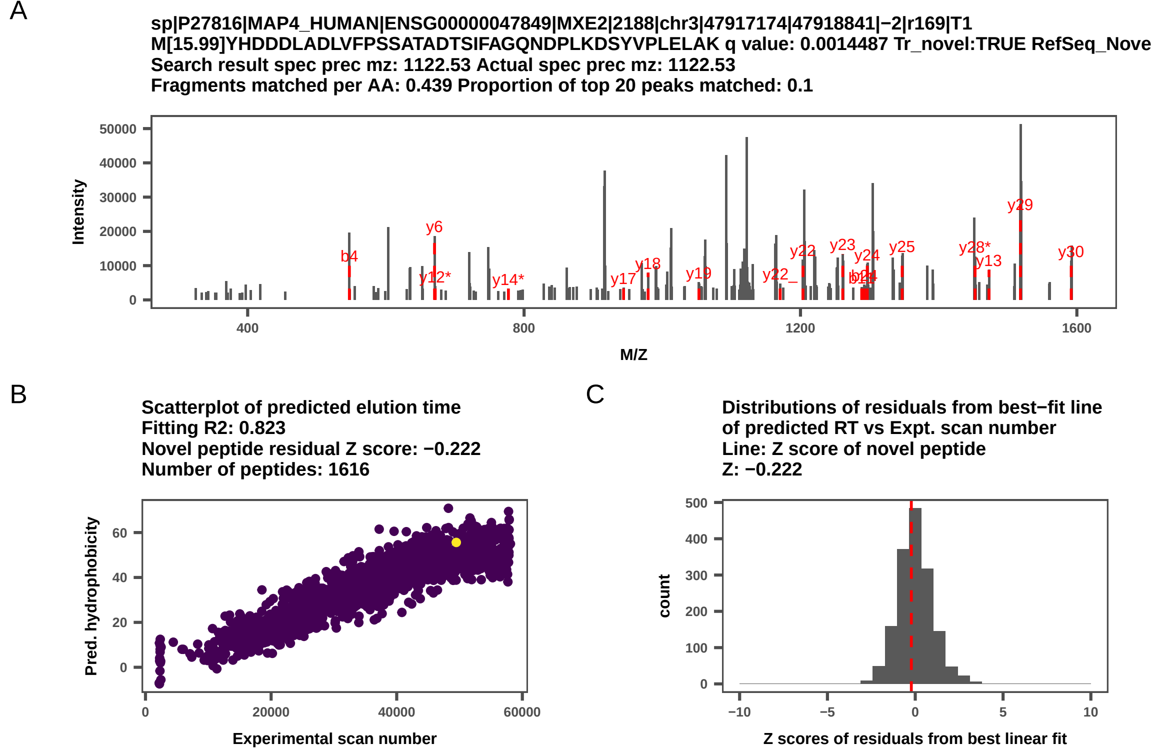
A
sp|P27816|MAP4_HUMAN|ENSG00000047849|MXE2|2188|chr3|47917174|47918841|−2|r169|T1
M[15.99]YHDDDLADLVFPSSATADTSIFAGQNDPLKDSYVPLELAK q value: 0.0014487 Tr_novel:TRUE RefSeq_Nove
Search result spec prec mz: 1122.53 Actual spec prec mz: 1122.53
Fragments matched per AA: 0.439 Proportion of top 20 peaks matched: 0.1
Intensity
M/Z
b4
y6
y12*
y14*
y17
y18
y19
y22_
y22
y23
y24
y25
y28*
y13
y29
y30
B
Scatterplot of predicted elution time
Fitting R2: 0.823
Novel peptide residual Z score: −0.222
Number of peptides: 1616
Pred. hydrophobicity
Experimental scan number
C
Distributions of residuals from best−fit line
of predicted RT vs Expt. scan number
Line: Z score of novel peptide
Z: −0.222
count
Z scores of residuals from best linear fit
